# Chemical Composition, Antipathogenic and Cytotoxic Activity of the Essential Oil Extracted from *Amorpha fruticosa* Fruits

**DOI:** 10.3390/molecules26113146

**Published:** 2021-05-24

**Authors:** Ioana Cristina Marinas, Eliza Oprea, Mihaela Buleandra, Irinel Adriana Badea, Bianca Maria Tihauan, Luminita Marutescu, Marin Angheloiu, Elena Matei, Mariana Carmen Chifiriuc

**Affiliations:** 1 Department of Botany and Microbiology, Faculty of Biology, Research Institute of the University of Bucharest-ICUB, University of Bucharest, 90-92 Panduri Street, 050663 Bucharest, Romania; ioana.cristina.marinas@gmail.com (I.C.M.); ciubuca.b@gmail.com (B.M.T.); lumi.marutescu@gmail.com (L.M.); carmen.chifiriuc@gmail.com (M.C.C.); 2 National Institute of Research & Development for Food Bioresources—IBA Bucharest, 5th Ancuta Baneasa Street, 020323 Bucharest, Romania; marian.angheloiu@sanimed.ro; 3 Department of Organic Chemistry, Biochemistry and Catalysis, Faculty of Chemistry, University of Bucharest, 90-92 Panduri Street, 050663 Bucharest, Romania; 4 Department of Analytical Chemistry, Faculty of Chemistry, University of Bucharest, 90-92 Panduri Street, 050663 Bucharest, Romania; mihaelabuleandra@yahoo.com (M.B.); irinel_badea@yahoo.com (I.A.B.); 5 National Institute of Materials Physics–Magurele, 405A Atomistilor Street, 077125 Măgurele, Romania; elena.matei@infim.ro; 6 Academy of Romanian Scientists, 3rd Ilfov Street, 051157 Bucharest, Romania

**Keywords:** essential oil, GC-MS, antimicrobial activity, antibiofilm activity, hydrophobicity, cytotoxicity, flow cytometry

## Abstract

The purpose of this paper was to characterize and investigate the antimicrobial potential of *Amorpha fruticosa* fruits essential oil (EO). The EO was extracted by hydrodistillation, analyzed by GC-MS, and then evaluated for its interaction with microbial and mammalian cells. The antimicrobial activity was assessed against bacterial and fungal strains, in a planktonic and adherent growth state, using qualitative and quantitative assays. The main components identified in *A. fruticosa* fruits EO were δ-cadinene, γ-muurolene, and α-muurolene. The Gram-positive strains proved to be more susceptible than Gram-negative bacteria and fungal strains. The EO exhibited good antibiofilm activity, inhibiting the microbial adherence to the inert (96-well plates and Foley catheter section) and cellular substrata. The flow cytometry analysis revealed as one of the possible mechanisms of antimicrobial action the alteration of cell membrane hydrophobicity. The cytotoxicity on the L929 cell line occurred at concentrations higher than 0.3 mg/mL. Taken together, our results demonstrate that *A. fruticosa* fruits EO contains active compounds with selective inhibitory effect on different microbial strains in planktonic and biofilm growth state, explained at least partially by the interference with microbial membranes due to their hydrophobic character.

## 1. Introduction

Nature has represented a rich source of medicinal compounds for thousands of years, an impressive number of modern drugs being derived from natural sources, especially of vegetal origin.

Desert false indigo (*Amorpha fruticosa* L.) is an invasive alien species native to southwest North America and naturalized in Europe as an ornamental and honey plant. Despite its beneficial role against erosion provided by an extensive root system, this species has an aggressive invasive character [1], forming monodominant communities replacing willow (*Salix triandra*) [2,3] and showing high allelopathic potential, as proven by its inhibitory effect on the initial development of oat, rapeseed, and sunflower [4]. Different ecological solutions have been proposed to simultaneously control its spread and exploit its positive potential, such as: obtaining nanocellulose from the *A. fruticosa* wood [5], extracting essential oil (EO) [6] from fruits, or exploiting by other green technologies [7]. 

The reported composition of EO extracted from the ripe fruit of *A. fruticosa* is highly diverse, but the common majority of compounds are sesquiterpenes, such as cadinenes and muurolenes [8,9,10,11,12]. It is generally accepted that EOs could represent an important alternative for fighting microbial infections, acting simultaneously on different microbial targets and exhibiting diverse mechanisms of antimicrobial action, such as degradation of the cell wall, cytoplasmic membrane lesions, coagulation of cytoplasm, depletion of proton motive force, proteins denaturation, etc. [13,14]. Due to their multi-component composition, EOs prevent the emergence of bacterial resistance compared to current antibiotics. The biological effects of *A. fruticosa* EO have been studied less. According to Ivanescu et al. (2014) [11], it has proved a moderate antibacterial activity on Gram-positive bacteria (*Staphylococcus aureus* and *Sarcina lutea*). 

Bacterial infections caused by medical device implantation account for over 50% of nosocomial infections [15]. The biofilm embedded bacteria exhibit the so-called phenotypic resistance to current biocides. This occurs through complex mechanisms that are not yet fully known, such as: increased cell density in the biofilm structure, increasing the metabolic processes yield and favoring the horizontal transfer of resistance genes, the impermeability of the extracellular matrix, the nutritional limitation, leading to a metabolic latency and a low growth rate, overexpression of resistance genes, especially those encoding efflux pumps, selection of a persister population, etc. [16,17]. On the other hand, the physico-chemical and pharmacological properties of an antibiotic are often not adequate for penetration into the biofilm matrix [18]. Therefore, the development of surfaces and materials with antimicrobial or antibiofilm effect is an important research direction for the biomaterials field [19]. 

Taking into account that the microbial cell wall hydrophobicity plays an important role in microbial adhesion to different surfaces, the EO strong hydrophobic character could alter the surface microbial cells by lipids fluidization, thus interfering with microbial adherence to different abiotic surfaces [20]. Therefore, the aim of this work was to evaluate the chemical composition, antipathogenic and cytotoxic activity of *Amorpha fruticosa* fruits EO and its ability to modulate the microbial cell wall hydrophobicity. Harnessing *A. fruticosa* fruits’ therapeutic potential to develop novel antimicrobial strategies could represent not only an economical solution but also a strategy for controlling the spread of this invasive plant species, thus protecting the local biodiversity. To our best knowledge, this is the first study that proves the antibiofilm activity of this species.

## 2. Results

### 2.1. Extraction of A. fruticosa Essential Oil

The average content of the *A. fruticosa* fruits in EO (7 determinations) was 0.43 ± 0.09% (mL EO/100 g fruits). The EO was colorless, with a specific odor. The medium density was 0.59 ± 0.02 g/mL.

### 2.2. GC-MS Analysis of Essential Oil

*A. fruticosa* fruits EO samples were analyzed by GC-MS to determine their phytochemical profile. A number of 24 volatile compounds were identified in *A. fruticosa* EO, representing 94.42% of the total area. The relative abundance, according to their elution order on a DB-5MS capillary column, was reported in Table 1 as a percentage from the total area obtained by GC-MS.

The most abundant chemical species were sesquiterpene hydrocarbons (82.74%), followed by oxygenated sesquiterpenes (7.37%), oxygenated monoterpenes (2.66%), and monoterpene hydrocarbons (2.15%). The main components of *A. fruticosa* fruits EO were: *δ*-cadinene (20.09%), *γ*-muurolene (12.79%), α-muurolene (12.54%), and *γ*-cadinene (7.86).

### 2.3. Antimicrobial Activity

The qualitative testing of the microbial susceptibility to the *A. fruticosa* fruits EO revealed the occurrence of growth inhibition zones for both reference and clinical *Staphylococcus aureus, Bacillus subtilis*, *Pseudomonas aeruginosa, Escherichia coli, Klebsiella pneumoniae, Acinetobacter baumannii, Enterococcus faecalis,* and *Candida albicans* strains. For the bacterial strains, the growth inhibition diameters have also been given for antibiotics, tested in accordance with the Clinical Laboratory Standards Institute (CLSI) recommendations. For fungal strains, the international standards do not recommend the disk diffusion method. Although the tested antibiotics proved to exhibit a better activity as compared to the tested EOs, a comparison is very difficult, as the antibiotic disk charge the diffusion rate in the culture medium and the mechanisms of action are different from those of the EOs. The MIC varied between 1.84 and 7.38 mg/mL for the Gram-positive strains and was in the range of 14.75–29.50 mg/mL for the Gram-negative ones. For the clinical strains isolated from urinary, vaginal, and respiratory tract infections (Table 2), the MIC values ranged between 7.38–29.50 mg/mL, while for reference, susceptible strains were between 3.69–29.50 mg/mL. The EO was more active against *S. aureus* reference and methicillin-resistant (MRSA) strains. The microbial adhesion to the inert substrate was mainly inhibited in the case of the Gram-positive bacterial strains, the Gram-negative *E. coli* O_126_B_16_ and for some of the yeast strains (*C. albicans* ATCC 101103 and *C. albicans* 393), at concentrations ranging from 0.92–7.38 mg/mL.

The strains that have been proven susceptible to the studied EO and to linalool (used as reference compound) were further selected to determine the influence of the EO on the preformed biofilms (Table 2).

#### 2.3.1. Eradication of Preformed Biofilms on Inert Substrates

The EO concentrations corresponding to fivefold MICs (MICx5) proved to eradicate all 24 h preformed biofilms adhered on inert substrates, exhibiting an inhibition index of biofilm growth (IIBG) between 61.24 and 94.97% (Table 3). When increasing the time of microbial biofilm formation, a decreasing ability of the EO to eradicate the biofilm was noticed, except for the monospecific biofilm formed by the MRSA 1263 strain. In the case of the Gram-positive strains, the MICx3 and MICx5 proved to be effective in eradicating the 24 h, preformed biofilms. The DMSO solvent did not interfere with the activity of the EO against 24 h and 48 h preformed biofilms (*p* > 0.05). The MIC and MBEC concentrations, although effective against planktonic cells were too low to eradicate the preformed biofilms.

**Figure 1:**
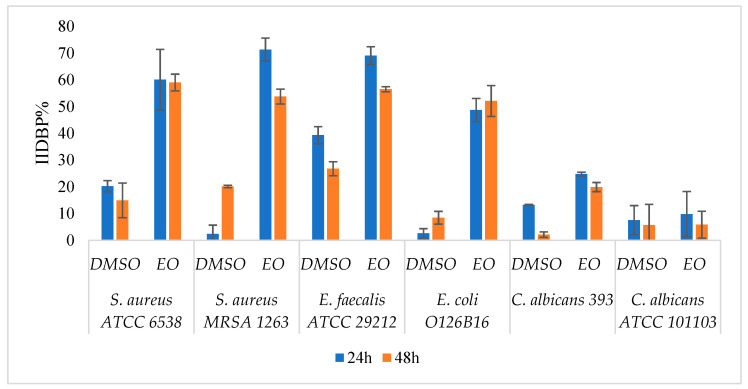
Graphic representation of IIDBP% determined after detachment of microbial sessile cells from the 24 h and 48 h preformed biofilms in vitro on Foley catheters treated with essential oil (EO) at MICx3 and DMSO (*p* < 0.05 for all strains, except *C. albicans* ATCC 101103; IIDBP = Inhibition Index Development for Preformed Biofilm; EO = essential oil; DMSO = dimethyl sulfoxide; MICx3 = increased three-time minimum inhibitory concentration).

The in vitro study of the influence of *A. fruticosa* fruits EO on the development of monospecific microbial biofilms on Foley catheters sections demonstrated the partial eradication of the preformed biofilms at EO concentrations corresponding to MICx3 values, the EO being especially active on *S. aureus* and *E. faecalis* biofilms (Figure 1). The DMSO solvent did not interfere with the antibiofilm activity of the EO (*p* < 0.05).

**Figure 2:**
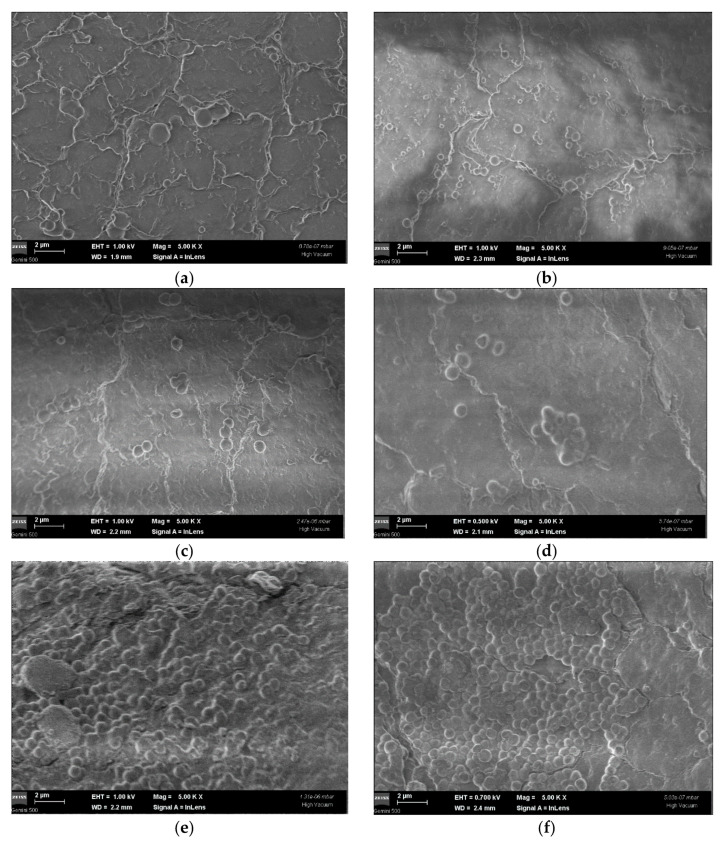
Micrographs of *S. aureus* and *C. albicans* cells/biofilms adhered to the surface of Foley catheters obtained by SEM. (**a**) *S. aureus* biofilm treated with EO, MICx3; (**b**) *C. albicans* biofilm treated with EO, MICx3; (**c**) *S. aureus* biofilm treated with DMSO; (**d**) *C. albicans* biofilm treated with DMSO; (**e**) *S. aureus* control strain; (**f**) *C. albicans* control strain. Magnification: 5000×; bars = 2 μm (MICx3 = increased three-time minimum inhibitory concentration; EO = essential oil; DMSO = dimethyl sulfoxide).

As shown in Figure 2, a lower adherence capacity of *S. aureus* and *C. albicans* on the catheter surface was evidenced at the SEM examination (5000×) of 48 h preformed biofilms. Additionally, the morphology and size of the adherent cells embedded in the biofilm were altered by the EO treatment. The untreated cells appeared to be regular in size, with a smooth spherical shape, suggesting the integrity of the cell envelope. After exposure to EO at MICx3 for 24 h, the morphology of the cells was modified, with the occurrence of smaller size cells, varying from 429–920 nm/580–885 nm in cases of positive controls (*C. albicans/S. aureus*) to 433–995 nm/520–930 nm (in cases of DMSO treated *C. albicans*/*S. aureus*), and respectively to 254–680/180–1448 nm after EO treatment of *C. albicans*/*S. aureus* cells. 

**Figure 3:**
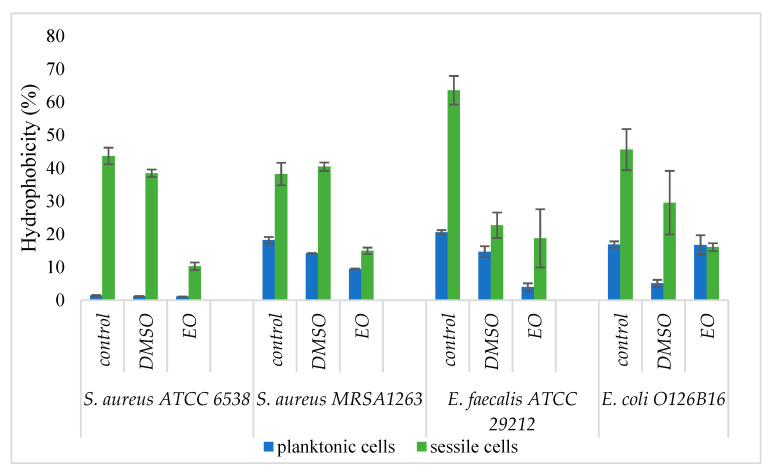
Graphic representation of the cell surface hydrophobicity of planktonic and sessile bacterial cells, positive control and treated with essential oil (EO) or DMSO, respectively (*p* < 0.05 for all strains; EO = essential oil; DMSO = dimethyl sulfoxide).

The measurement of the absorbance of the microbial cultures in the hydrophilic phase after one hour of contact with the organic layer (isooctane) revealed that the EO drastically decreases the hydrophobicity of sessile cells and of planktonic Gram-positive strains (Figure 3). These results suggest the biofilm dispersing properties of the tested EO and its promising potential as an antibiofilm agent.

#### 2.3.2. Flow Cytometry Evaluation of the Potential Antifungal Mechanisms 

The PI staining suggested that the *Candida* spp. cells are viable even after 24 h in the presence of the EO, indicating its fungistatic effect. Thus, subinhibitory concentrations were used for NR staining to observe possible changes in intracellular lipids. 

**Figure 4:**
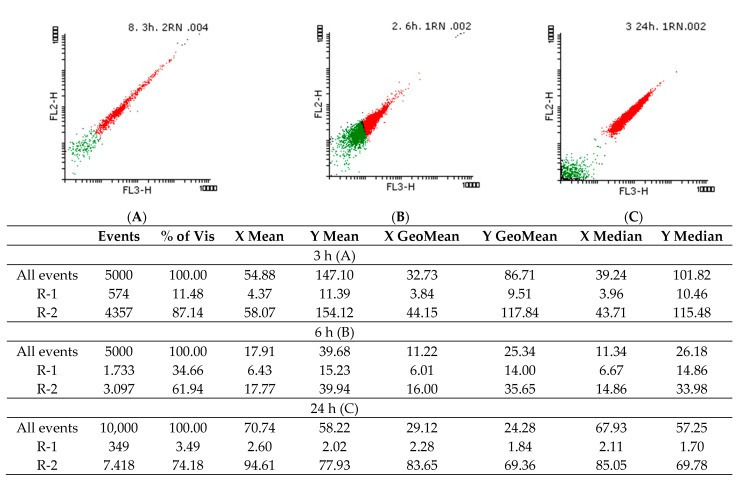
Density plots of *C. famata* 945 cells stained with NR treated with 2.95 mg/mL *A. fruticosa* fruits EO in temporal dynamics (R1–green: live cells and R2–red: dead cells) after 3 (**A**), 6 (**B**), and 24 h (**C**) of contact with strains (FL = fluorescence parameter (the fluorescent signal emitted for neutral lipids labeled (580 nm) was detected on FP-2, and for polar lipids (610 nm) on FL-3).

**Figure 5:**
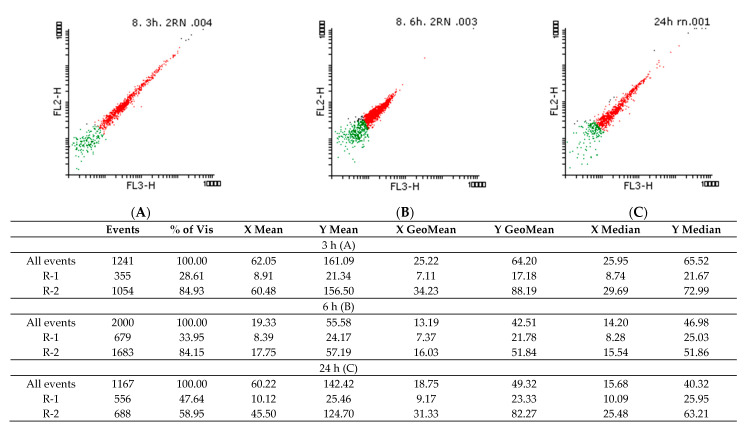
Density plot and histograms of *C. albicans* 393 cells stained with NR treated with 2.95 mg/mL of *A. fruticosa* fruits EO in temporal dynamics (R1–green: live cells and R2–red: dead cells) after 3 (**A**), 6 (**B**) and 24 h (**C**) of contact with strains (FL = fluorescence parameter (the fluorescent signal emitted for neutral lipids labeled (580 nm) was detected on FP-2, and for polar lipids (610 nm) on FL-3).

**Figure 6:**
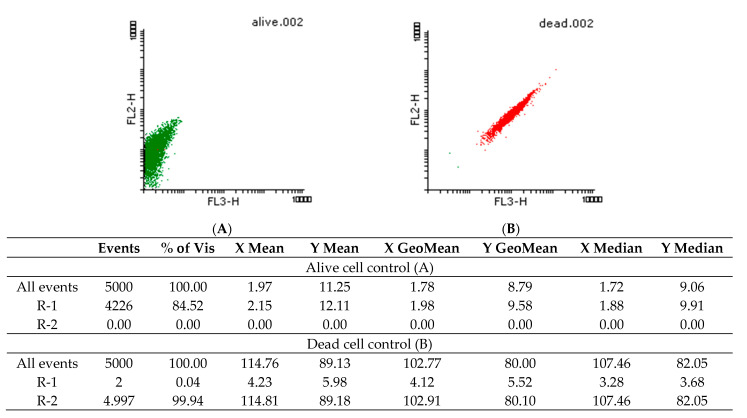
Density plots of yeast cells stained with NR for live (**A**) and dead (**B**) cells–positive and negative control (R1–green: live cells and R2–red: dead cells; FL = fluorescence parameter (the fluorescent signal emitted for neutral lipids labeled (580 nm) was detected on FP-2, and for polar lipids (610 nm) on FL-3).

Figure 4 and Figure 5 show the density graphs and the histograms obtained for the NR staining of yeast cells treated with 2.95 mg/mL *A. fruticosa* fruits EO. In the first three hours (A), a shock is observed, after which the cells recovered, but after 24 h (C), the intracellular lipids are fluidized by the tested EO. The cells appeared dead, but according to the data obtained by PI labeling, the fungal cells are still viable after 24 h. In Figure 6, the controls for living and dead cells are highlighted.

### 2.4. Assessment of Human Cells Viability and EO Cytotoxicity

**Figure 7:**
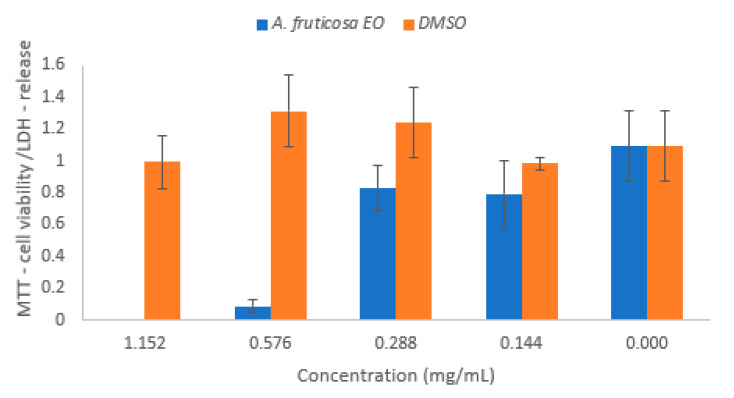
The concentration-dependence of cell viability (MTT) and LDH release for L929 cell line treated with *A. fruticosa* fruits EO and DMSO for 24 h (LDH = lactate dehydrogenase; MTT = 3-(4,5-dimethylthiazol-2-yl)-2,5-diphenyltetrazolium bromide; EO = essential oil; DMSO = dimethyl sulfoxide).

The influence on human cells was investigated by MTT and LDH assays on L929 cells co-cultured with *A. fruticosa* fruits EO and DMSO (Figure 7).

The mitochondrial activity, assessed with the MTT test, has been inhibited by *A. fruticosa* fruits EO. The tested EO has increased the LDH release in a reproducible dose-dependent manner. The cytotoxic effect occurred at concentrations higher than 0.3 mg/mL.

### 2.5. Influence of A. fruticosa Fruits EO on Microbial Ability to Adhere to Mammalian Cells

In the presence of *A. fruticosa* fruits EO, the microbial adhesion index to mammalian cells ranged between 57.30% and 79.73%, the most intense inhibitory effect being noticed in the case of *E. coli* strain at a concentration of MIC/4 (Table 4).

## 3. Discussion

The multiple targets and mechanisms of the antimicrobial activity of EOs are well known [21,22]. So far, the available literature data regarding the content of *A. fruticosa* fruits EO highlighted a high variation from 0.32% [12] to 1.8% [11], the results probably being influenced by the harvesting time, geographical area, physiological maturity, and storage conditions. It is well known that the variability of the chemical composition is correlated with different biological properties. Our results regarding the chemical composition of *A. fruticosa* fruits EO are similar to a Bulgarian study [12], proving that the geographic origin of the plants has a significant impact. According to Ivănescu et al. (2014) [11], the EO color was yellow, while we obtained a colorless one, exhibiting better antimicrobial activity. Fewer but more concentrated compounds were identified by GC-MS, which could explain the more pronounced antimicrobial properties. The major compounds were *δ*-cadinene (20.09%), *γ*-muurolene (12.79%), *α*-muurolene (12.54%), and *γ*-cadinene (7.86%). Compared to the data published by Georgiev et al. [12], we obtained a higher concentration of the *δ*-cadinene and a lower one for *γ*-cadinene. A recent paper identified 22 components in the *A. fruticosa* EO, with the main compounds caryophyllene (17.64%), *α*-guaiene (14.70%), and *γ*-muurolene (5.98%). Moreover, Kozuharova et al. have reported the naphthalene presence (6.75%) that was undetected in our samples [23]. Other studies have shown similar results regarding the presence of δ-cadinene and γ-muurolene as major constituents of the *A. fruticosa* fruits EO [9,12].

Due to its strong hydrophobic nature, the EO was solubilized in DMSO to facilitate the homogenous mixture with the culture media before assessing the antimicrobial properties. However, the DMSO solvent did not interfere with the antimicrobial activity of the EO on the tested strains. The qualitative testing of the antimicrobial properties of the EO revealed a potent inhibitory effect, especially on the Gram-positive bacterial strains (*S. aureus, B. subtilis,* and *E. faecalis*), the obtained results being similar to those reported by Ivanescu et al. (2014) [11]. The *δ*-cadinene and α-muurolene are probably contributing to the antimicrobial effect, taking into account that the *Xenophyllum poposum* EO, containing the two compounds has also shown antibacterial activity against *S. aureus* [24]. An explanation for the most potent effect against Gram-positive strains could be the particular structure of the Gram-negative cell wall, harboring an additional layer represented by the outer membrane, which does not allow for hydrophobic molecules’ entrance as readily as the Gram-positive bacterial envelope [25]. The alcoholic monoterpenes citronellol and linalool were also reported to possess antimicrobial activity. Citronellol presented a rapid bactericidal effect against *E. coli* [26]. Linalool was found to inhibit 17 of 18 strains (including Gram-positive cocci and rods and Gram-negative rods) [27]. Delaquis [28] also demonstrated the inhibitory effect of linalool against Gram-negatives. There is evidence for the antimicrobial activity of some sesquiterpenes from *A. fruticosa* EO, such as germacrene D, known for its valuable antibacterial and antifungal activities [29] as well as *γ*-muurolene, that could be responsible for the antimicrobial activity against *B. subtilis* and *C. tropicalis* (including clinical strains) of the *Piper ovatum* Vahl EO [30]. Another sesquiterpene, *β*-caryophyllene, was active against 24 from 32 tested strains isolated from do dental plaque, at concentrations up to 100 mg mL^−1^ [31]. Caryophyllene oxide was found to be active against *S. aureus* and *Proteus mirabilis* [32], while T-cadinol was found to be active toward *S. aureus* (CMI 24 μg/mL) and *Trichophyton mentagrophytes* (CMI 24 μg/mL). Moreover, the TEM analysis showed that T-cadinol interacted with the cell envelope of *S. aureus*, causing bacterial lysis and subsequent fatal loss of the intracellular material, being thus considered a bacteriolytic compound [33]. The antibiofilm activity of linalool has been already demonstrated [34,35] and it is confirmed in part in the present study. The activity against the fungal strains was very low, but however, the flow cytometry assay highlighted that the tested EO, at a concentration ranging between 29.50 and 2.95 mg/mL, was fungistatic and solubilized the intracellular lipids, without affecting fungal cells viability. This result correlates with the observed decrease of the hydrophobicity of the cell surface and implicitly of the adhesion capacity to biotic and abiotic substrates, that are facilitated by hydrophobic interactions.

The *A. fruticosa* fruits EO inhibited the microbial adherence on the inert substrate, which represents the first stage of the infectious process, but only for antibiotic susceptible strains. 

Considering that microbial biofilms have a much lower sensitivity than planktonic cells, higher concentrations corresponding to MICx3 and MICx5 values were used to determine the antibiofilm effect of the EO on preformed biofilms.

For the evaluation of the antibiofilm effect on medical devices, Foley catheter sections were used for the development of monospecific biofilms. Microbial adhesion to various surfaces is an extremely complex process, mediated by physicochemical nonspecific interactions, such as Lifshitz-van der Waals, electrostatic forces, acid-base interactions, and Brownian motion forces [36]. In biological systems, hydrophobic interactions are usually the strongest of all non-covalent bonds, and these types of interactions often mediate adhesion to various inert surfaces. Hydrophobicity has been shown to play an important role in a wide range of microbial infections. Microbial hydrophobicity is defined by the energy of attraction between nonpolar or slightly polar cells in an aqueous phase and can be evaluated by several methods [37]. In our study, the chosen method was MATH (Microbial Adhesion to Hydrocarbon), in which the aqueous phase was phosphate-buffered saline (PBS) and the organic phase isooctane. 

It was observed that IIDBP% is inversely proportional to hydrophobicity, which means that the less hydrophobic cell surface, the lower the adhesion to medical devices. Due to the hydrophobic nature of EO compounds, these can interact with the lipid bilayer of the outer or cytoplasmic membranes [25], modifying the hydrophobic character of cell surfaces.

For Gram-positive strains, it has been shown that with increasing antibiotic resistance, the cell surface is more hydrophobic, and, according to Lather et al. (2016), the content in lipoteichoic acids is higher [38]. In our study, it has been observed that planktonic MRSA cells have a more hydrophobic character than the reference methicillin-susceptible *S. aureus* strain. Previous studies demonstrated that the interaction with the EOs, mainly terpenes, caused an alteration of cell surface hydrophobicity [20].

Rubini et al. (2021) [39] aimed to obtain materials with an enhanced hydrophilic surface to prevent biofilms formation. This desideratum could be achieved by incorporating an EO into various materials to ensure a controlled release in a lower than cytotoxic concentration for modulating the hydrophobicity of the microbial cell surface and decreasing its adherence and biofilm development ability. The optimal concentration regarding cytotoxicity and antibiofilm activity for the tested EO was 0.3 mg/mL. This concentration didn’t exhibit a microbicidal effect, but it beneficially modified the microbial cells envelope so as to inhibit the adhesion to the medical device surfaces and human cells.

## 4. Materials and Methods

### 4.1. Plant Material

The *A. fruticosa* fruits were collected from Scrovistea forest, Romania in late August 2014. Their taxonomic affiliation was confirmed, and voucher specimens were deposited in the herbarium of the Botanical Garden “Dimitrie Brândză” from the University of Bucharest (No. 401256). The plants were manually sorted and dried at room temperature.

### 4.2. Chemicals and Reagents

The culture medium, PBS, DMSO, linalool, and penicillin/streptomycin were supplied by Sigma, Milano, Italy. MTT solvent and regent were from Roche Diagnostics GmbH, Mannheim, Germany. All other reagents were of analytical grade.

### 4.3. Extraction of A. fruticosa Fruits Essential Oil

An amount of 100 g of air-dried *A. fruticosa* fruits was ground by passing through a 100-mesh sieve and then subjected for 4 h to water distillation using a Clevenger-type apparatus. The EO concentration has an expression of mL EO/100 g fruits. The obtained EO was stored at +4 °C until tested and analyzed [40]. 

### 4.4. GC-MS Analysis of Essential Oil

Samples of EOs were diluted in hexane (1:100) and volumes of 1 μL were injected (splitless mode) and analyzed with a Thermo Electron system (Polaris Q ion-trap MS combined with a Focus GC and a Triplus Autosampler), equipped with a DB-5MS capillary column (25 m × 0.25 mm; 0.25 μm of film thickness). Helium (1 mL/min) was used as a carrier gas. The selected GC program was: initial temperature 60 °C for 3 min, an increase of 10 °C/min up to 200 °C and to 240 °C at 12 °C/min and held for 10 min at 240 °C. The detector operated in positive electron impact mode (70 eV), in the range of m/z 35–300, full scan mode. All peaks of the chromatograms were analyzed using the Xcalibur^®^ software and NIST 11 Mass Spectral Library, the identification being carried out with a minimum similarity level of 80% [41]. Alkane standard solution for GC (C_8_-C_20_ in hexane) was used for retention indexes (RI) calculation [42]. The relative percent of individual components was calculated based on GC peak areas.

### 4.5. Antimicrobial Activity Assay

#### 4.5.1. Microbial Strains

For testing the antimicrobial activity, there were used both reference (8 strains) and clinical isolated strains (5 strains), either Gram-positive (*S. aureus* ATCC 6538, MRSA 1263, *B. subtilis* 12488, *B. subtilis* ATCC 6633 *E. faecalis* ATCC 29212) or Gram-negative (*P. aeruginosa* ATCC 27853, *P. aeruginosa* 134202, *E. coli* ATCC 25922, *E. coli* O126B16, *K. pneumoniae* ATCC 134202, *K. pneumoniae* 11–urinary tract infection, *A. baumannii* 77 sc–respiratory tract infection) bacteria, as well as yeasts (*C. famata* 945-respiratory tract infection, *C. famata* CMGBy.14, *C. utilis* 15–urinary tract infection, *C. albicans* ATCC 101103, *C. albicans* 393–vaginal infection). 

#### 4.5.2. Antimicrobial Activity against Planktonic Cells

The stock solutions of *A. fruticosa* fruits EO used for further assays were prepared 1:1 in DMSO (dimethyl sulfoxide). The antimicrobial activity screening was determined by employing an adapted disk diffusion technique, using 10 μL of stock solution of EO, which contained 295 mg EO solubilized in 1 mL of DMSO. The minimum inhibitory concentrations (MICs) were measured as described previously [43]. Briefly, serial dilutions of the stock solutions in liquid medium (Brain heart infusion broth for bacterial strains and Sabouraud broth for yeasts) were prepared in 96 wells plates (concentrations 29.5–0.46 mg/mL). Then 10 μL of the microbial suspension with the standard density of 0.5 Mc Farland was added to each well, positive and negative controls were used for each strain. The positive control antibiotic disks used in diffusion method include oxacillin (1 µg) (Bio-Rad, Hercules, CA, USA); ticarcillin-clavulanic acid (75 + 10 µg) (Bio-Rad); ceftriaxone (30 µg) (Bio-Rad); cefalexin (30 µg) (Bio-Rad); vancomycin (30 µg) (Bio-Rad); ofloxacin (5 µg) (Bio-Rad); colistin (10 µg) (Bio-Rad); erythromycin (15 μg) (Bio-Rad). The antibiotics were chosen according to CLSI and literature data for each tested strain. The plates were incubated for 24 h at 37 °C. The absorbance was measured at 620 nm with an Apollo LB 911.

#### 4.5.3. The Influence of *A. fruticosa* Fruits EO on the Microbial Adherence Capacity to the Inert Surface

The influence on the ability of microbial adherence to the inert substratum (96-well plate, untreated polystyrene) was measured after running the quantitative analysis of the antimicrobial activity, through the microtiter method, by evaluating the biofilm biomass, after fixation with cold methanol (5 min) and 1% crystal violet staining (for 15 min). The optical density of the biological material resuspended in acetic acid 33%, stirred 150 rev/min for 15 min was determined by reading the absorbance at 490 nm [44]. Negative and positive controls were used. The positive control highlights the sterile working conditions, where no microbial cell has adhered to the substrate. Positive controls are represented by the natural adhesion of untreated microbial strains.

#### 4.5.4. The Influence of *A. fruticosa* Fruits EO on Microbial Biofilms Developed on Inert Surface

The 24 h and 48 h microbial biofilms were formed into 96-well sterile plates (untreated polystyrene) as follows: 100 μL of culture medium was added to each well (TSB -tryptic soy broth for bacteria, Sabouraud for yeast) and inoculated with 10 μL of standard 0.5 McFarland microbial inoculum and then, the plates were incubated for 24 h and 48 h at 37 °C, respectively. After every 24 h, the plates were washed three times in PBS for removal of the non-adherent cells. A fresh medium containing EO concentrations corresponding to the MICx3 and MICx5 values was added. The plates were incubated for 24 h at 37 °C [45,46]. The *slime* production test was used to evaluate the adhered biomass. The method was performed in duplicate. The Inhibition Index Development for Preformed Biofilm (IIDPB) was calculated as follows:

IIDPB% = 100 − (A_s_ × 100)/A_c_ (1)

where A_s_ = the absorbance of the biofilm formed and treated with EO/DMSO and A_c_ = the absorbance of the biofilm formed untreated. 

#### 4.5.5. The Influence of *A. fruticosa* Fruits EO on Preformed Microbial Biofilms on Foley Catheter Sections

The development of the microbial biofilm of 24 h and 48 h was achieved on Foley catheter sections of 4 mm × 5 mm (length × width) size, sterilized by UV for 20 min. The protocol was adapted from Anghel et al., 2013 [47] with few modifications. Briefly, the catheters were seeded with 200 μL microbial inoculum (0.5 McFarland) and were incubated for 24 h and 48 h at 37 °C. These were washed in PBS, moved in a fresh medium after each 24 h and further incubated at 37 °C. After pre-formation of microbial biofilm, the catheters were treated with MICx3 of EO and incubated for 24 h at 37 °C. Catheters were washed to remove the planktonic cells, placed in 1 mL PBS and vortexed for 5 min for detachment of the biofilm. Successive ten-fold dilutions were made with the formed suspension and spotted in agar media plates for CFU (colony forming unit)/mL determination. The method was performed in duplicate. The Inhibition Index Development for Preformed Biofilm was calculated as follows:

IIDPB% = (M − E) × 100)/M(2)

where: M = lg CFU/mL for strain control and E = lg CFU/mL for sample. 

The adherence capacity of strains on the catheters’ surface was analyzed by scanning electron microscopy (SEM) using Gemini 500 equipment from Zeiss (Oberkochen, Germany). Before the morphological examination of adherent cells embedded in the biofilm, the catheters have incubated for 48 h at 37 °C were washed with sterile distilled water and fixed with methanol (either those treated or untreated with EO). Additionally, the catheters’ morphology was analyzed as such by SEM, prior to microbial cell adhesion.

#### 4.5.6. Influence of *A. fruticosa* Fruits EO on the Hydrophobicity of Sessile and Planktonic Bacterial Cells 

Comparative assessment of cell surface hydrophobicity for planktonic and sessile cells, as well as changes induced by the studied EO, was performed according to [48,49] Gogra et al. (2010) and Qiao et al. (2012) with some modifications. EO concentrations corresponding to MIC/4 values at a final volume of 1 mL were used to determine the influence on planktonic surface cells. The standard 0.5 McFarland microbial inoculum was seeded with 200 µL and incubated for 24 h at 37 °C. Foley catheters (dimensions 4 mm × 5 mm: length × width, sterilized for 20 min with UV radiation) were used for 24 h biofilm development. The catheters were washed with PBS, placed in fresh medium, and incubated for 24 h at 37 °C. After incubation, the washed catheters were reintroduced in culture medium with MIC values of EO and incubated for 24 h at 37 °C. Catheters were washed, placed in 1 mL PBS and vortexed for 5 min to detach the biofilm. The sessile and planktonic cells were previously washed 3x with PBS (centrifugal program: 10 min, 10,000 rpm), the pellets were taken up in PBS and vortexed for 120 s. Turbidity was adjusted to 2 McFarland and the absorbance was read at 600 nm (A_0_). 0.5 mL of isooctane was to microbial suspension and vortexed for 120 s. The samples were left to stand for 60 min (till two phases are completely separated), the organic phase is taken over and the absorbance at λ = 600 nm (A_1_) of the aqueous phase is read. Strain, solvent, and sterility controls were used for each strain. The method was performed in duplicate. The hydrophobicity of microbial cell surface (H) was expressed as a percentage of optical density remaining in the organic phase:

H% = (1 − A_1_/A_0_) × 100 (3)

#### 4.5.7. Flow Cytometry Evaluation of Viability and Intracellular Lipids Modification Using Propidium Iodide (PI) and Nile Red (NR) Staining

To determine the influence of *A. fruticosa* EO on the microbial membrane lipids, concentrations of 29.5–2.95 mg/mL were tested in temporal dynamics, i.e., after 3, 6, and 24 h of contact with *Candida* spp. strains. The fungal cells were washed 3 times, resuspended in phosphate buffer saline (PBS), and labeled with Propidium Iodide (PI) and Nile Red (NR). The fluorochromes concentration was 5 µL/mL, and the staining protocol was applied at room temperature for 10 min before data acquisition using the FACS Calibur cytometer. PI, which absorbs at 495 nm and emits at 637 nm (FL-3), binds specifically to nucleic acids and highlights the dead cells [50]. NR fluorochrome was used for lipids-specific staining, the fluorescent signal emitted for neutral lipids labeled (580 nm) being detected on FL-2, and for polar lipids (610 nm) on FL-3 [51]. The Flowing Software 2 program was used for statistical analysis and interpretation of histograms. 

### 4.6. Assessment of Cell Viability and Cytotoxicity by MTT and LDH Assay 

L929 mouse fibroblasts (ECACC–*European Collection of Authenticated Cell Cultures*) were selected as a model for cytotoxicity assessment of *A. fruticosa* EO. L929 cells were cultivated in DMEM (Dulbecco’s Modified Eagle Medium) media supplemented with 10% FBS (Fetal Bovine Serum), and 1% Pen/Strep (penicillin /streptomycin solution, 50 µg/mL) for 24 h at 37 °C, 95% humidity with 5% CO_2_. After 24 h, cells were washed with PBS, harvested using trypsin, and counted using Trypan Blue and a hemocytometer. The seeding density for the MTT and LDH assays was optimized at 5 × 10^4^.

#### 4.6.1. MTT Assay

The MTT assay evaluates the cellular metabolic activity, therefore, it is a good indicator of cell viability, proliferation, and cytotoxicity. This colorimetric assay is based on the reduction of a yellow tetrazolium salt (3-(4,5-dimethylthiazol-2-yl)-2,5-diphenyltetrazolium bromide or MTT) to purple formazan crystals by metabolically active cells [52].

Cells seeded at 5 × 10^4^ density in a clear 96 well cell culture plate were treated with *A. fruticosa* fruits EO (concentrations: 1.152–0.144 mg/µL) and incubated for 24 h at 37 °C, 95% humidity with 5% CO_2_. After 24 h of exposure to *A. fruticosa* EO, cells were incubated for 4 h with MTT reagent at 37 °C, 95% humidity with 5% CO_2_. After incubation, cells were treated with MTT solvent for 15 min at room temperature. Absorbance was measured using FlexStation 3 (Molecular Devices Company, Sunnyvale, CA, USA) at OD = 570 nm.

#### 4.6.2. LDH Assay

The LDH assay involves the assessment of cell death by quantification of plasma membrane damage. This increase in the amount of enzyme activity in the supernatant directly correlates to the amount of formazan formed. Therefore, the amount of color formed in the assay is proportional to the number of lysed cells [53]. With the LDH Cytotoxicity Detection Kit (Roche), LDH activity was measured in culture supernatants using FlexStation 3 (Molecular Devices Company, Sunnyvale, CA, USA) at 492 nm with a 600 nm wavelength reference. Cells were prepared and treated alike as for the MTT assay. 

### 4.7. Influence of A. fruticosa Fruits EO on Microbial Ability to Adhere to Mammalian Cells

The adherence index of microbial strains treated with MIC/4 of EO to the L929 murine fibroblasts cells and the adherence pattern were established by the adapted Cravioto method [54]. Briefly, L929 murine fibroblasts cell monolayers (70–80% confluence) were washed with PBS and a fresh medium was added. PBS suspensions of bacterial strains were adjusted to 10^8^ CFU/mL and 1 mL was used for L929 murine fibroblasts cells inoculation. The inoculated plates were incubated for 2 h at 37 °C [55]. After incubation, the monolayers were washed 3 times with PBS, fixed in cold methanol, and stained with 1:10 v/v Giemsa solution for 20 min. The plates were washed, dried, and examined by optic microscopy (Axiolab (Zeiss) microscope) using wet objective (×2500 magnification) in order to evaluate the adherence indexes (the ratio between the number of the eukaryotic cells with adhered bacteria from 200 eukaryotic cells counted) and patterns. The negative control was mammalian cells without microbial strains, and the positive control was the untreated microbial cells suspension (without DMSO or EO). 

### 4.8. Statistical Analysis

Data were analyzed with statistical analysis computer software (Excel–Microsoft Office). The one-way ANOVA was used to compare the effects between EO and DMSO. Differences were considered significant when the *p*-value was less than 0.05. The level of significance was set at 5%.

## 5. Conclusions

Our results demonstrated that among other biological activities, the EO extracted from *A. fruticosa* fruits contains antimicrobial active compounds (such as linalool, citronellol, *β*-caryophyllene, caryophyllene oxide, *α*- and *γ*-muurolene, germacrene D, *δ*-cadinene, *τ*-cadinol) with moderate microbicidal activity on Gram-positive bacterial strains. The tested EO has successfully interfered with the microbial adhesion and biofilm development on inert and cellular substrates and decreased the cellular hydrophobicity. Therefore, the tested EO could be either incorporated in different biomaterials to render them resistant to microbial colonization or included in different controlled release systems to prevent the formation of microbial biofilms. One of the underlying mechanisms of the observed antibiofilm effect could be the decrease of intracellular lipids, affecting the hydrophobic character of microbial cell envelopes.

In an approach specific to the circular bioeconomy, the valorization of this invasive species by extracting useful essential oils from *A. fruticosa* could represent at the same time an ecological solution for the extensive spread of this plant. Besides its antimicrobial applications, *A. fruticosa* EO could represent a valuable source of *α*- and *γ*- muurolene, based on broadly accessible raw materials.

## Figures and Tables

**Table 1:** Chemical composition of *A. fruticosa* fruits EOs seen by GC-MS.

No	RT ^a^	RI ^b^	Compound	RA ^c^
1	7.85	1044	*trans-β*-Ocimene (*E*)	1.65
2	8.03	1055	*cis-β*-Ocimene (Z)	0.50
3	8.93	1108	Linalool	2.15
4	10.91	1263	Citronellol	0.51
5	12.73	1365	*α*-Cubebene	0.59
6	13.07	1390	*α*-Ylangene	2.35
7	13.12	1393	*α*-Copaene	6.46
8	13.60	1431	*α*-Gurjunene	0.94
9	13.75	1443	*β*-Caryophyllene	6.25
10	13.86	1451	*β*-Gurjunene (calarene)	1.24
11	14.01	1462	*α*-Guaiene	1.98
12	14.19	1477	Humulene (*α*-caryophyllene)	1.22
13	14.43	1496	*γ*-Muurolene	12.79
14	14.53	1504	Germacrene D	0.45
15	14.67	1516	*α*-Amorphene	4.77
16	14.72	1520	*α*-Muurolene	12.54
17	14.92	1537	*γ*-Cadinene	7.86
18	15.01	1544	*δ*-Cadinene	20.09
19	15.19	1560	Cadinadiene-1,4	1.87
20	15.28	1567	*α*-Calacorene	1.34
21	15.84	1615	Caryophyllene oxide	0.63
22	16.35	1660	*γ*-Eudesmol	1.35
23	16.45	1669	*τ*-Cadinol	0.93
24	16.62	1684	*α*-Eudesmol	4.46
			Monoterpene hydrocarbons	2.15
			Oxygenated monoterpenes	2.66
			Sesquiterpene hydrocarbons	82.74
			Oxygenated sesquiterpenes	7.37
			Total	94.92

^a^ RT = retention time (min); ^b^ RI = Kovats index, measured relative to n-alkanes (C_8_–C_20_) on a DB-5MS capillary column; ^c^ RA = relative area (%) = relative contents expressed as percentages of the total oil composition.

**Table 2:** The MIC and MBEC values of the *A. fruticosa* fruits EO.

Strains	DI ^a^ (mm)		MIC ^b^ (mg/mL)			MBEC ^c^ (mg/mL)		
	EO ^d^	ATB ^e^	EO ^d^	L ^f^	DMSO ^g^	EO ^d^	L ^f^	DMSO ^g^
*S. aureus* ATCC 6538	13.00 ± 1.41	9.00 ± 0.00 ^h^	3.69	2.48	>29.50	1.84	1.24	>29.50
*S. aureus* MRSA 1263	7.50 ± 0.71	28.00 ± 0.00 ^i^	1.84	9.91	>29.50	0.92	4.96	>29.50
*B. subtilis* 12488	8.50 ± 0.71	17.33 ± 0.58 ^j^	7.38	4.96	7.38	0.92	0.31	1.84
*B. subtilis* ATCC 6683	11.50 ± 0.71	20.50 ± 2.12 ^j^	7.38	-	7.38	0.92	-	1.84
*E. faecalis* ATCC 29212	9.50 ± 0.71	27.50 ± 2.10 ^i^	7.38	9.91	>29.50	3.69	2.48	29.50
*P. aeruginosa* ATCC 27853	7.00 ± 0.00	13.25 ± 1.26 ^k^	14.75	4.96	29.50	14.75	2.48	14.75
*P. aeruginosa* 134202	-	18.33 ± 1.53 ^k^	29.50	˃9.91	>29.50	29.50	-	29.50
*K. pneumoniae* 11	7.00 ± 0.00	13.67 ± 1.15 ^l^	>29.50	2.48	>29.50	>29.50	-	>29.50
*K. pneumoniae* ATCC 134202	7.50 ± 0.71	12.50 ± 0.71 ^l^	29.50	4.96	29.50	14.75	2.48	14.75
*E. coli* ATCC 13202	-	24.50 ± 6.36 ^m^	>29.50	2.48	>29.50	>29.50	0.62	>29.50
*E. coli* O_126_B_16_	7.50 ± 0.71	9.00 ± 0.00 ^n^	14.75	9.91	>29.50	14.75	4.96	29.50
*A. baumannii* 77 sc	7.00 ± 0.00	19.00 ± 0.00 ^p^	29.50	0.62	29.50	29.50	-	29.50
*C. famata* 945	-	-	14.75	˃9.91	14.75	14.75	-	14.75
*C. albicans* 393	8.00 ± 0.00	-	7.38	0.62	14.75	7.38	0.31	14.75
*C. utilis* 15	-	-	>29.50	0.62	>29.50	>29.50	0.31	>29.50
*C. famata* CMGBy.14	-	-	>29.50	1.24	>29.50	>29.50	0.62	>29.50
*C. albicans* ATCC 101103	± ^r^	-	7.38	1.24	14.75	0.92	-	7.38

^a^ DI = diameter of growth inhibition; ^b^ MIC = minimum inhibitory concentration; ^c^ MBEC = minimum biofilm eradication concentration on inert substrate; ^d^ EO = essential oil; ^e^ ATB = antibiotic; ^f^ L = linalool; ^g^ DMSO = dimethyl sulfoxide corresponding concentration with EO; ^h^ oxacillin; ^i^ erythromycin; ^j^ vancomycin; ^k^ ceftriaxone; ^l^ cefalexin; ^m^ ofloxacin; ^n^ ticarcillin -clavulanic acid; ^p^ colistin; ^r^ microbial colonies growth inside the diameter of inhibition. For the fungal strains, the international standards do not recommend the assessment of antifungal activity by the disk diffusion method.

**Table 3:** Inhibition of preformed microbial biofilms of 24 and 48 h induced by *A. fruticosa* fruits EO.

IIBG% ^a^		MICx3 ^b^ (mg/mL)		MICx5 ^c^ (mg/mL)	
		EO ^d^	DMSO ^e^	EO ^d^	DMSO ^e^
*S. aureus* ATCC 6538 *	24 h	71.05 ± 0.84	48.88 ± 5.35	80.12 ± 1.9	51.32 ± 4.56
	48 h	73.35 ± 0.98	17.94 ± 3.68	77.57 ± 1.51	37.21 ± 1.86
*S. aureus* MRSA 1263 *	24 h	76.49 ± 1.98	43.15 ± 3.25	77.11 ± 7.05	46.25 ± 5.62
	48 h	79.39 ± 1.17	38.65 ± 2.06	86.38 ± 3.05	48.24 ± 14.11
*E. faecalis* ATCC 29212 *	24 h	84.09 ± 5.42	42.09 ± 4.43	94.97 ± 6.8	62.61 ± 10.67
	48 h	48.21 ± 4.30	8.31 ± 5.79	92.17 ± 9.79	47.51 ± 3.70
*E. coli* O_126_B_16_ *	24 h	83.46 ± 4.67	52.13 ± 2.79	82.52 ± 10.32	60.11 ± 0.38
	48 h	76.67 ± 2.57	59.64 ± 0.59	77.96 ± 2.93	64.92 ± 3.68
*C. albicans* 393 *	24 h	69.00 ± 9.51	41.27 ± 5.21	77.39 ± 2.84	50.58 ± 2.60
	48 h	76.93 ± 1.95	57.40 ± 3.05	75.44 ± 6.1	65.37 ± 2.62
*C. albicans* ATCC 101103	24 h	61.95 ± 0.49	62.94 ± 3.32	80.12 ± 1.9	66.99 ± 3.42
	48 h	55.13 ± 6.94	56.37 ± 2.59	77.57 ± 1.51	61.55 ± 2.04

^a^ IIBG = inhibition index of biofilm growth; ^b^ MICx3 = increased three-time minimum inhibitory concentration; ^c^ MICx5 = increased five-time minimum inhibitory concentration; ^d^ EO = essential oil; ^e^ DMSO = dimethyl sulfoxide corresponding concentration with EO; * *p* < 0.05.

**Table 4:** The anti-adherence activity of *A. fruticosa* fruits EO.

Strains	*S. aureus* ATCC 6538	MRSA 1263	*E. faecalis* ATCC 29212	*E. coli* O_126_B_16_
AICS ^a^ (%)				
EO ^b^	70.08	79.73	61.96	57.30
DMSO ^c^	100	100	96.25	98.70
Positive control ^d^	99.54	100	98.70	100
AP ^e^				
EO	1	3	2	1
DMSO	3	3	1	2
Positive control ^d^	3	3	1	2

^a^ AICS = adherence index to cellular substrate (the ratio between the number of the eukaryotic cells with adhered bacteria from 200 eukaryotic cells counted); ^b^ EO = essential oil; ^c^ DMSO = dimethyl sulfoxide; ^d^ Positive control = the untreated microbial cells suspension (without DMSO or EO); ^e^ AP = adherence pattern (1–localized; 2–diffuse; 3–aggregative).

## Data Availability

Not applicable.

## Sample Availability

Samples of the essential oil are available from the corresponding authors.
